# Variation in optimal communication modes during neonatal resuscitation

**DOI:** 10.1016/j.resplu.2026.101308

**Published:** 2026-03-29

**Authors:** Heidi M. Herrick, Anne M. Ades, Preston B. Cline, Daniel A. Dworkis

**Affiliations:** a 3401 Civic Center Blvd, 2nd Floor Main Building, Department of Pediatrics, Division of Neonatology, Children’s Hospital of Philadelphia, Philadelphia, PA 19104, USA; b 1011 Bay Ridge Ave, Suite #221, Mission Critical Team Institute, Annapolis, MD 21403, USA; c 1500 San Pablo St, Department of Emergency Medicine, Keck School of Medicine of USC, Los Angeles, CA 90033, USA

**Keywords:** Communication, Resuscitation, Neonatology

## Abstract

**Background:**

Effective communication is paramount for neonatal resuscitation. Best practices emphasize clear, coordinated team communication. High-performing teams use both routine and critical communication modes, adjusting their approach as acuity evolves. However, the degree to which interprofessional neonatal intensive care unit (NICU) teams share an understanding of when each mode is optimal is unknown.

**Methods:**

We conducted a cross-sectional study of NICU providers participating in a team-development day at a large children’s hospital. After an instructional session defining routine and critical communication, participants reviewed a hypothetical unplanned extubation and cardiac arrest scenario including 14 steps split into pre-code, code, and post-code phases. Participants rated optimal mode of team communication for each step using a 5-point Likert scale. Consensus was defined as ≥60% agreement on communication mode for each step. We compared physicians and non-physicians across scenario phases.

**Results:**

Forty-eight providers participated, representing physicians, nurses, advanced practice providers, and respiratory therapists. Participants reached consensus on all code phase steps, selecting critical communication. No consensus was achieved for pre-code or post-code. Physicians selected more critical communication than non-physicians during the pre-code phase (median 4.0 vs 3.0, *p* = 0.001) with no differences during code or post-code.

**Conclusion:**

Providers within a cohesive NICU team showed divergent mental models regarding optimal communication during early and late phases of a hypothetical resuscitation. This heterogeneity may hinder rapid team alignment and represent an important target for quality-improvement efforts. Future work should evaluate communication during real resuscitations and develop strategies to support shared communication expectations across roles.

## Introduction

Communication between members of a clinical team is critical to effective resuscitation.^1^, ^2^, ^3^ The Neonatal Resuscitation Program (NRP), Pediatric Advanced Life Support (PALS), and Advanced Cardiovascular Life Support (ACLS) programs all emphasize the importance of effective communication to enhance team performance and patient outcomes during resuscitation.^4^, ^5^, ^6^ Furthermore, the Joint Commission identified communication as a frequent root cause of sentinel events across all healthcare disciplines, including >70% of perinatal deaths and injuries.^7^, ^8^

Prior work on medical team communication identified two distinct modes of communication used by high-performing teams that operate in and out of emergencies, called routine and critical communication.^9^ Routine communication uses open ended questions, modulated tones, and engaged body language to build connections and deeper understanding among team members. By contrast, critical communication uses short, direct statements, a projected voice, and assertive body language for efficient and directive transfer of information. Routine communication is for normal operations and team building, while critical communication is for completing urgent tasks. Teams switch between these communication modes as they move between high and low acuity moments during a resuscitation. The degree to which team members agree or disagree on the best communication mode during parts of a resuscitation is unknown and may affect team cohesion and performance.

Using an established, high-functioning neonatology intensive care unit (NICU) team as a test model, we hypothesized team members would disagree on the best communication mode at different resuscitation points. In this manuscript, we present the analysis of team-level agreement on communication mode during a hypothetical resuscitation.

## Methods

### Study design and population

We performed a cross-sectional analysis on a convenience sample of neonatology-trained providers working within a neonatology network centered at a major US children’s hospital. Participants included interprofessional representation from leadership and front-line NICU providers who attended a team-development day. During the day, participants attended a 90-minute session on routine and critical communication that combined didactics with collaborative inquiry, allowing participants to discuss what routine and critical communication looked like in their environment. Didactics defined the concepts of routine and critical communication and provided examples during resuscitation as demonstrated in Table 1. Participants then completed a survey rating best communication mode for the steps of a hypothetical NICU resuscitation. The survey did not specify a resuscitation algorithm, and both NRP and PALS are used within the network. The IRB deemed this study exempt from IRB oversight.

**Table 1:** Characteristic, purpose, and examples of routine versus critical communication.^9^

**Communication mode**	**Characteristics**	**Purpose**	**Example**
Routine	– Open-ended questions – Conversational tone – Engaged body language	Promotes shared understanding, situational awareness, & team cohesion during lower-acuity periods	“How is the baby responding to CPAP?” “Should we prepare intubation equipment?”
Critical	– Short, direct statements – Projected voice – Assertive body language – Closed-loop communication	Enables rapid coordination and execution of urgent tasks during high-acuity moments	“Heart rate 50—start chest compressions now.” “Administer 0.01 mg/kg Epinephrine and state when it is complete.”

**Fig. 1:**
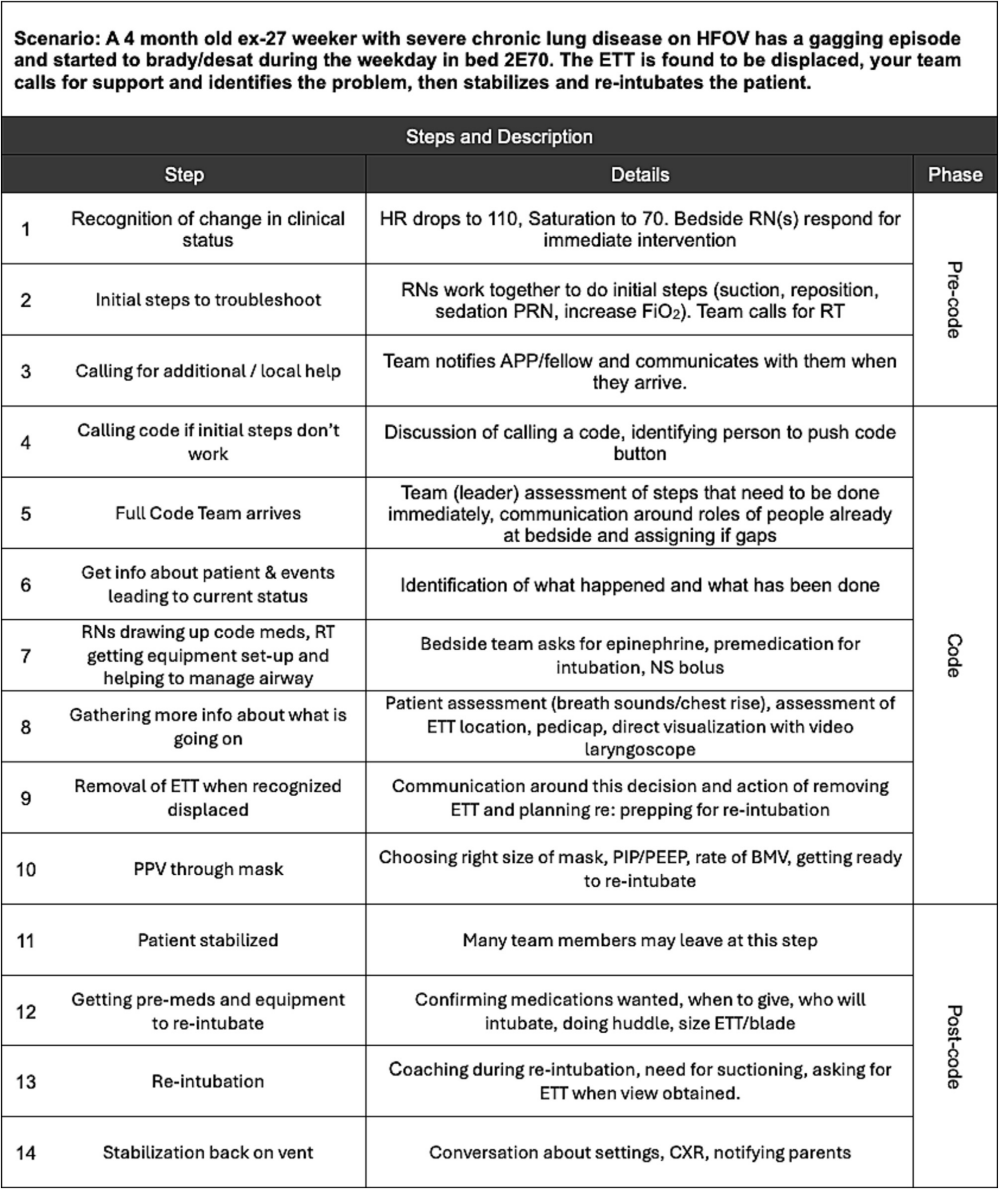
**Clinical scenario**. Clinical scenario developed by neonatal experts and explored by the study participants. The “Step Name” column lists the 14 steps for which participants rated the best mode of communication, and the “Details” column lists relevant details further explaining each step. The “Phase” column denotes the phase of the resuscitation, pre-code, code, and post-code. Of note, the “phase column” was not displayed during the participants’ ranking of communication style as to not bias participants. HFOV: high-frequency oscillatory ventilation; brady/desat: bradycardia/desaturation; ETT: endotracheal tube; HR: heart rate; RN: registered nurse; PRN: as needed; FiO_2_: fraction of inspired oxygen; RT: respiratory therapist; APP: advanced practice provider; NS: normal saline; PPV: positive pressure ventilation; PIP: peak inspiratory pressure; PEEP: positive-end expiratory pressure; BMV: bag mask ventilation; CXR: chest x-ray.

Neonatology experts on our team developed a hypothetical NICU scenario of an unplanned extubation and subsequent decompensation leading to cardiac arrest. The scenario involved 14 steps, each with a distinct action and supporting details, divided into three phases: pre-code (steps 1–3), code (steps 4–10), and post-code (steps 11–14). Fig. 1 shows the scenario and lists the steps and associated details.

For each step, participants rated the best mode of team communication along a five-point Likert scale ranging from “*Routine*” at one, to “*Critical*” at five. Intermediate values reflected increasing urgency and directive language, two- routine-leaning communication, three- communication in either style, and four- critical-leaning communication. Participants were also asked their professional role: physician (MD), nurse (RN), advanced practice provider (APP), or respiratory therapist (RT).

### Statistical analysis

We defined consensus on best communication mode by 60% agreement on the Likert scale for that step.^10^ We explored variation by professional role across the three phases of the scenario using a Mann Whitney U Test with an alpha of 0.05. We employed Bonferroni correction for multiple comparisons, yielding an effective alpha of 0.007. Sample size necessitated the division of participants into physician and non-physician categories. Data analysis was performed in R, extended by the “tidyverse” package.^11^, ^12^

## Results

A total of 48 individuals participated in the study: 21 MDs (43.8%), 15 RNs (31.3%), six APPs (12.5%), and five RTs (10.4%). One participant (2.1%) did not report their role. Of the 48 participants, 44 (91.7%) rated best communication mode for all resuscitation steps, and four (8.3%) rated 13 of 14 steps.

Overall, participants rated the scenario as requiring more critical than routine communication with a median average rating across all steps of 4 (interquartile range (IQR) 3–5). Participants rated communication during the code phase (steps 4–10) as the most critical (median 5, IQR 4–5), compared to communication during either the pre-code (steps 1–3) or post-code (steps 11–14) phases (median 4, IQR 3–4 for both).

**Fig. 2:**
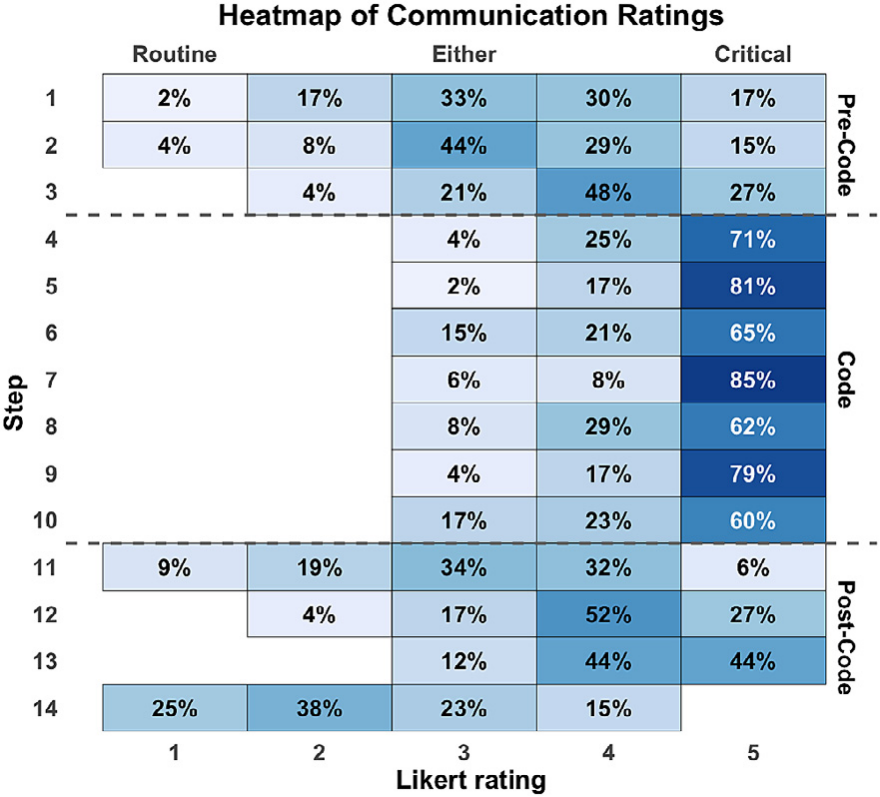
**Heatmap of communication ratings across all participants**. Heatmap distribution of participant’s choice of best mode of communication across all steps of the clinical scenario. In this figure, each “row” of the heatmap corresponds to a step of the clinical scenario, and each “column” corresponds to a rating on the communication-based Likert scale which ranged from “*Routine”* at 1 to “*Critical”* at 5. Intermediate values reflected increasing urgency and directive language, two- routine-leaning communication, three- communication in either style, and four- critical-leaning communication. Each cell shows the percentage of participants who chose that mode of communication for that step. Darker color shading corresponds to higher percentages of agreement. Horizontal dashed lines mark the boundaries between the three phases of the scenario (Pre-Code, Code, and Post-Code).

Using a consensus threshold of >60%, the team agreed critical communication to be the best communication mode for all 7 code phase steps. Pre-code and post-code stabilization phases showed more variability in best communication mode and did not reach consensus. Fig. 2 shows a heatmap of responses by step across all participants and highlights the stronger levels of agreement and more critical communication during the code phase as opposed to pre- or post-code phases. Comparing the distributions of communication scores across phases showed significant differences between the code phase and either pre- or post-code phase (*p* < 0.001 for both comparisons), but no significant difference between pre-code and post-code phases (*p* = 0.31).

**Fig. 3:**
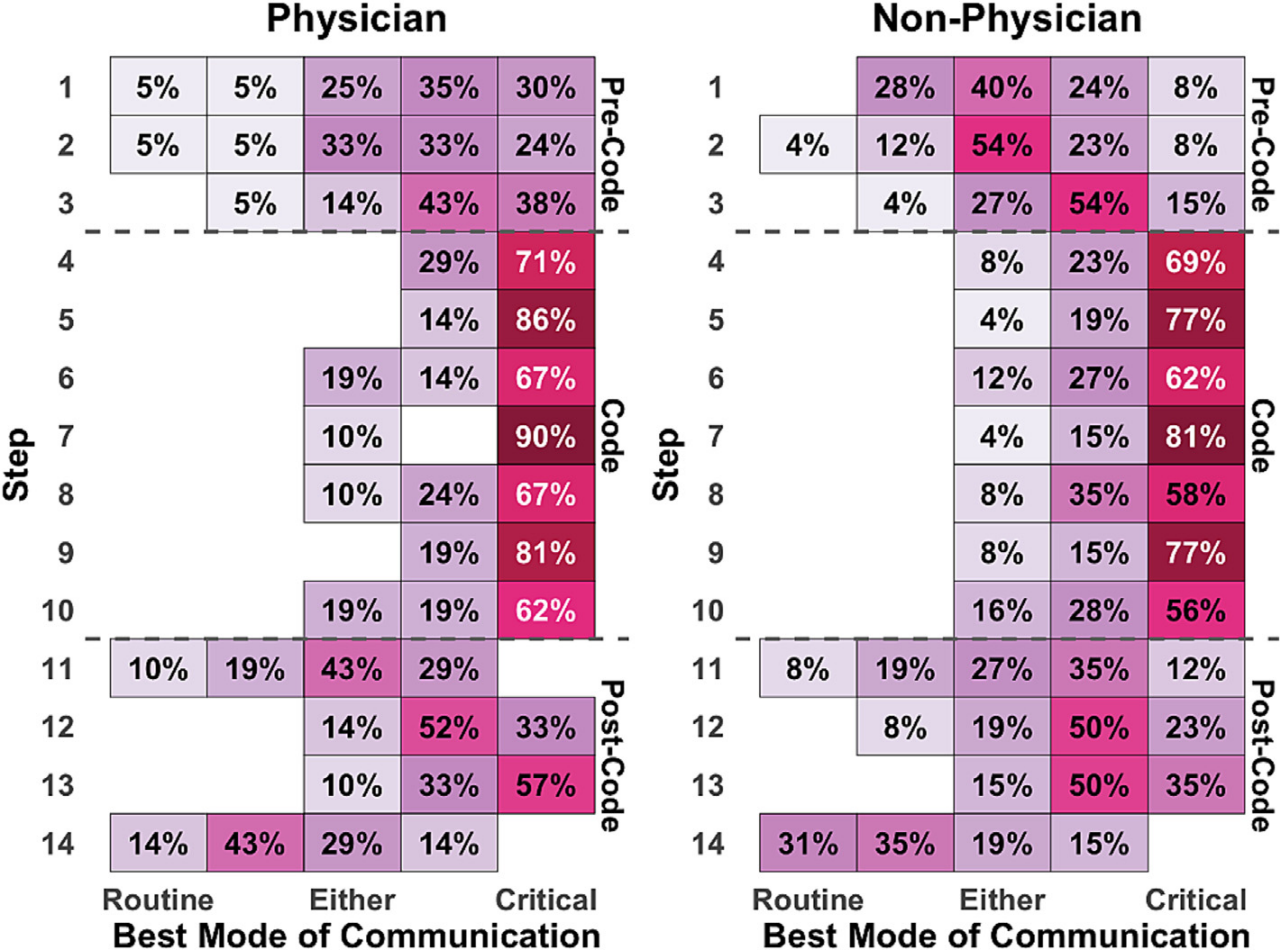
**Comparison of physician and non-physician communication choices**. Heatmap distributions of participant’s choice of best mode of communication across all steps of the clinical scenario, split by primary role into Physician (left) and Non-Physician (right). In this figure, each “row” of the heatmap corresponds to a step of the clinical scenario, and each “column” corresponds to a rating on the communication-based Likert scale which ranged from “*Routine”* at 1 to “*Critical”* at 5. Intermediate values reflected increasing urgency and directive language, two- routine-leaning communication, three- communication in either style, and four- critical-leaning communication. Each cell shows the percentage of participants who chose that mode of communication for that step. Darker color shading corresponds to higher percentages of agreement. Horizontal dashed lines mark the boundaries between the three phases of the scenario (Pre-Code, Code, and Post-Code).

We explored the association between provider role and communication mode, dividing the cohort into physician (*n* = 21, 43.8%) and non-physician providers (*n* = 26, 54.2%). Physicians favored more critical communication during the pre-code phase as compared to non-physicians (median 4, IQR 3–5 versus 3, IQR 3–4, *p* = 0.001 even after adjusting for multiple comparisons). No significant differences were observed between physicians and non-physicians during code or post-code phases. Across the entire case, physicians also favored more critical communication (median 5, IQR 3–5 compared to 4, IQR 3–5, *p* = 0.02), though this did not remain significant after adjusting for multiple comparisons. Fig. 3 shows the comparison of heatmaps of communication choices between these two groups, highlighting pre-code phase differences.

## Discussion

In this single-center study of NICU team opinion on communication during an unplanned critical event, we found substantial heterogeneity in perceived best communication mode in both pre-code and post-code phases. Additionally, we found differences in the perceived best communication mode in the pre-code phase between physicians and non-physicians. To our knowledge, this is the first study to explicitly evaluate team-level understanding of routine vs critical communication in a medical setting.

While participants agreed critical communication to be the best communication mode during the code phase, these results indicate their mental models for what “best communication looks like” during the early and late parts of the case were mixed. Since different mental models of best communication during high-stakes clinical operations can impact team performance under pressure, these results suggest a potential barrier to effective neonatal resuscitation through increasing risk of miscommunication, even in this well-established, high-functioning NICU team.

Additionally, after dividing participants into physicians and non-physicians, we found significant differences in how these groups thought about communication during the beginning of their team response to the clinical scenario. During both expected and unexpected acute clinical events, the beginning of a critical response is often the most challenging, chaotic, and uncertain phase, while also being the most important phase for setting the tone for the resuscitation.^13^, ^14^ Differences in expected modes of communication during this initial response might be especially challenging for team dynamics and performance. To address this, future studies need to explore the disagreements we found between physicians and non-physicians with larger sample sizes to explore potential differences by professional role (e.g. RT versus RN).

The etiology of these different mental models about communication is unclear. Heterogeneity might come from differences in how individuals perceive urgency and consequence within a given moment, profession-based differences in training on team communication, or factors specific to the scenario we chose or the team within which we tested it. Further work is needed to explore the origin of this heterogeneity and how to best address it.

We will highlight two key limitations. First, this study asked volunteer NICU providers to consider a hypothetical situation rather than observing their communication during an actual clinical event. The observed patterns in communication “as imagined“ may be different from communication “as done” during a real event. Second, the limited sample size of some professions precluded further between-group analysis other than physicians versus non-physicians.

While assessing work-as-done is preferred to work-as-imagined to evaluate system performance,^15^^,^^16^ this study demonstrated lack of consensus even for imagined ideal communication which would have otherwise gone unnoticed. The within team and across-profession heterogeneity in best communication modes during acute NICU events represents an important target for further research around shared mental models of communication. Future work should focus on developing and training teams in different communication models and shared principles guiding which model to use when, as well as evaluating communication patterns observed in actual NICU resuscitations.

## Conclusion

Heterogeneity on best communication mode during hypothetical acute neonatal events exists within team and across professions. Future work is needed to study communication modes during clinical resuscitations and developing consensus communication modes.

## Funding sources

HMH is supported by an Agency for Healthcare Research and Quality career development grant (K08HS029029).

## CRediT authorship contribution statement

**Heidi M. Herrick:** Writing – review & editing, Writing – original draft, Methodology, Conceptualization. **Anne M. Ades:** Writing – review & editing, Methodology, Conceptualization. **Preston B. Cline:** Writing – review & editing, Methodology. **Daniel A. Dworkis:** Writing – review & editing, Writing – original draft, Methodology, Formal analysis, Data curation, Conceptualization.

## Declaration of competing interest

The authors declare that they have no known competing financial interests or personal relationships that could have appeared to influence the work reported in this paper.
